# Effects of Physical Activity Interventions on Health Outcomes among Older Adults Living with HIV: A Systematic Review and Meta-Analysis

**DOI:** 10.3390/ijerph19148439

**Published:** 2022-07-11

**Authors:** Mi-So Shim, Dabok Noh

**Affiliations:** 1 Mo-Im Kim Nursing Research Institute, College of Nursing, Yonsei University, Seoul 03722, Korea; misoshim1111@gmail.com; 2 College of Nursing, Eulji University, Seongnam-si 13135, Korea

**Keywords:** HIV, older adults, exercise, physical activity, meta-analysis

## Abstract

There is a lack of evidence regarding the effects of exercise on older individuals living with HIV. This systematic review and meta-analysis examined previous studies on physical activity interventions for people living with HIV aged ≥50 years. The effectiveness of the interventions on various physical and psychological health outcomes was evaluated. Databases used for this review included PubMed, EMBASE, CINAHL, and Cochrane Library CENTRAL. Twelve randomized controlled trials on physical activity interventions for people ≥50 years and living with HIV were included. Standardized mean differences were calculated using random-effect models. All effect sizes were expressed using Cohen’s *d* values and their 95% confidence intervals (CIs). Physical activity interventions had a significant effect on walking capacity (Cohen’s *d*: 0.467; 95% CI [0.069, 0.865]). The effect sizes on cardiorespiratory fitness, weight, and health-related quality of life were not significant. These findings suggest that physical activity interventions for people living with HIV aged ≥50 years are effective for the improvement of walking capacity. Further larger and higher-quality studies are required to determine the full effects of physical activity interventions on various health outcomes among older adults with HIV.

## 1. Introduction

With the introduction and development of antiretroviral therapy in the mid-1990s, life expectancy for people living with human immunodeficiency virus (HIV) infection increased [1,2]. People living with HIV can survive into their early 70s if they maintain medication adherence and a healthy lifestyle [1]. However, the incidence of geriatric syndromes, such as urinary incontinence, slow gait, and sensory deficits, is increased in people living with HIV compared with uninfected people [3]. According to the Joint United Nations Program on HIV/AIDS (UNAIDS), the number of people living with HIV aged ≥50 years increased globally from 5.8 million in 2015 to an estimated 8.5 million in 2020 [4]. Moreover, 50% of the people living with HIV in the United States are reportedly ≥50 years old [3]. A cohort study conducted in the Netherlands predicted that the proportion of people living with HIV who are ≥50 years old will reach 70% by 2030 [5].

People living with HIV experience various health problems related to aging. HIV-induced changes in immune activation and inflammatory response promote aging even when HIV is suppressed with antiretroviral therapy [6]. In addition, the toxicity of antiretroviral treatment affects the individual’s chronic low-grade inflammatory phenotype [7]. Because of these immune change mechanisms, the incidence rates of cardiovascular disease, liver disease, bone disease, kidney disease, cancer, and cognitive decline have been reported to be much higher in people living with HIV than in uninfected people [6,8]. Mental health problems among people living with HIV are also prevalent and severe [9,10]. For the prevention of physical and psychological comorbidities of older people living with HIV, Erlandson and Karris (2019) recommended assessing various health outcomes and improving modifiable risk factors [11]. Thus, it is important to observe modifiable health outcomes related to the major comorbidities of people living with HIV, such as cardiorespiratory fitness, walking capacity, metabolic parameters including lipid profile and blood glucose, body composition and weight, and depression [11,12,13].

Physical activity has been suggested as an important component of interventions in improving the physical and mental health of older adults living with HIV [8,11]. The health benefits of physical activity are numerous and physical activity levels among people living with HIV have been reported to be lower than in patients with other chronic diseases [14,15]. Results from studies on physical activity interventions for older adults living with HIV have reported that physical activity has positive effects on cardiorespiratory fitness [16,17,18], walking capacity [17,19], depression, and health-related quality of life [19]. Therefore, it is necessary to evaluate the effectiveness of physical activity interventions on physical and psychological health outcomes in older adults living with HIV by integrating the findings of randomized clinical trials (RCTs).

Recent systematic reviews and meta-analyses have reported that exercise is effective for enhancing various health outcomes in people living with HIV. Specifically, exercise improves cardiovascular parameters [20], lipid profiles and blood glucose [21], CD4 count [22], and depression and anxiety [23,24]. However, there is a lack of evidence regarding the importance of exercise for supporting the health of specific populations of people living with HIV, such as older adults and women [24]. Thus, the purpose of this review was to evaluate the effects of physical activity interventions on the health outcomes among people living with HIV aged ≥50 years.

## 2. Materials and Methods

### 2.1. Design

The systematic review and the meta-analysis were guided by the Cochrane Handbook for Systematic Reviews of Interventions [25]. The reporting of this study conforms to the Preferred Reporting Items for Systematic Reviews and Meta-Analyses (PRISMA) 2020 Checklist [26]. The protocol of this review was registered online with the International Prospective Register of Systematic Reviews (PROSPERO; No. CRD42022304641).

### 2.2. Eligibility Criteria

The inclusion criteria in this review were as follows: (a) studies on people living with HIV; (b) studies with participants either entirely aged ≥50 years or with an average age of at least 50 years; (c) studies about interventions to improve physical activity; (d) studies that included a comparison group; (e) studies assessing physical and psychological health outcomes; (f) RCTs; and (g) articles written in English. Studies were excluded if they did not specify the age of the participants. Non-randomized experimental studies, study protocols, reviews, editorials, conference proceedings, national and international reports, and grey literature were also excluded.

### 2.3. Data Sources and Search Strategy

We searched MEDLINE, EMBASE, CINAHL, and the Cochrane Library CENTRAL for all articles published prior to 17 November 2021. Details of the search strategy are provided in Supplementary Materials Table S1.

### 2.4. Selection Process

First, duplicate articles were removed from among the studies that were identified through the searches. Two independent researchers initially screened the titles and abstracts of all articles. Then, the researchers assessed the full-text articles and excluded those that did not meet the inclusion criteria while recording the reasons for exclusion. The final articles were selected through discussion between reviewers.

### 2.5. Data Extraction

Two independent researchers extracted data from the included studies using a data extraction form and disagreements were resolved by discussion. The characteristics of the study (author, publication year, country where the study was conducted, study design), participants (target population, age, sex, sample size), intervention (type of intervention, mode of delivery, duration, frequency, providers of the intervention, comparison condition, intervention adherence), health-related outcomes, and results were included in the data extraction form. When two papers were published from one study, all the data from each of the papers were extracted. The research team sent emails to corresponding authors for requesting data for the three articles that did not present all the values required for our meta-analyses [16,17,27]; however, because we did not receive the data, we only used the studies with available data for meta-analyses.

### 2.6. Risk-of-Bias Assessment

The two researchers independently assessed the risk-of-bias using the Cochrane risk-of-bias tool for randomized trials, version 2 [28], and reached a consensus through discussion. The tool includes the following five domains of bias: (a) bias arising from the randomization process, (b) bias due to deviations from intended interventions, (c) bias due to missing outcome data, (d) bias in measurement of the outcome, and (e) bias in selection of the reported result. The response options to the signaling questions listed under each domain of bias included: “yes”, “probably yes”, “probably no”, “no”, and “no information”. The risk-of-bias for each domain was judged as “low”, “having some concerns”, or “high”, based on the answers to the signaling questions. The overall risk-of-bias for each study was classified as being “low”, “having some concerns”, or “high”, based on the judgments of the five individual domains.

### 2.7. Effect Measures and Synthesis Methods

The included studies that reported sufficient numerical data to calculate effect sizes were synthesized quantitatively in meta-analyses. The meta-analyses were conducted on outcomes for which data from at least three studies could be synthesized. We used a random-effects model from the DerSimonian and Laird method [29] to calculate the overall effect. The effect size was based on the standardized mean difference. We computed Cohen’s *d* effect sizes and their 95% confidence intervals (CIs). The Cohen’s *d* effect size was interpreted as follows: <0.20, small; 0.20–0.79, medium; ≥0.80, large [30]. Statistical heterogeneity was assessed using the I-squared statistic value (0–40%: possible unimportance; 30–60%: moderate heterogeneity; 50–90%: substantial heterogeneity; and 75–100%: considerable heterogeneity) [31]. In addition, sensitivity analyses were conducted by excluding single studies one by one from the meta-analyses to assess robustness of the synthesized results. The publication biases in the meta-analyses were analyzed by visual inspection of funnel plots and statistically calculation using Egger’s test [32]. The meta-analyses were conducted using Comprehensive Meta-Analysis Version 3 (Biostat, Englewood, NJ, USA).

## 3. Results

### 3.1. Study Selection

**Figure 1:**
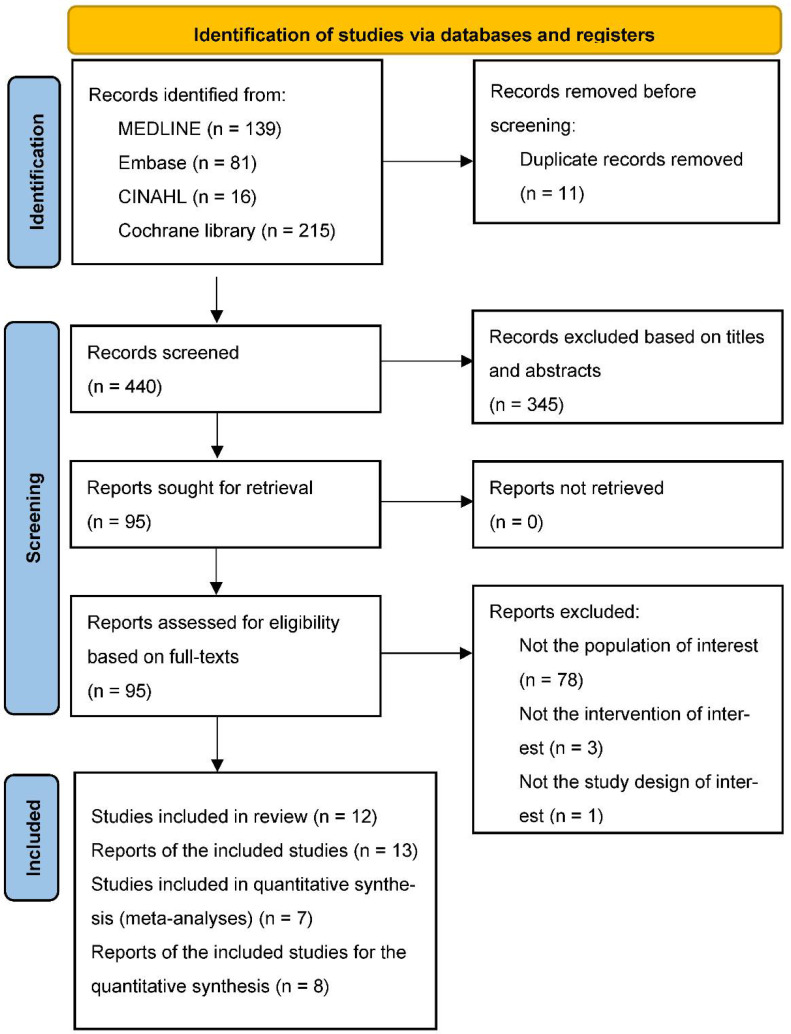
Flow diagram of the study selection process according to the PRISMA Guideline.

After duplicate articles were removed, 440 articles remained. Of these, 345 articles were excluded following a review of titles and abstracts. Full-text assessments were performed on the remaining 95 articles for eligibility, of which 82 were excluded: 78 articles did not include people living with HIV aged ≥50 years, 3 did not provide physical activity interventions, and 1 was not an RCT. As a result, 13 articles derived from 12 studies were included in this review; among them, 8 articles derived from 7 studies were included in the quantitative analysis. A PRISMA flowchart outlining the study selection process is shown in Figure 1.

### 3.2. Risk-of-Bias

**Figure 2:**
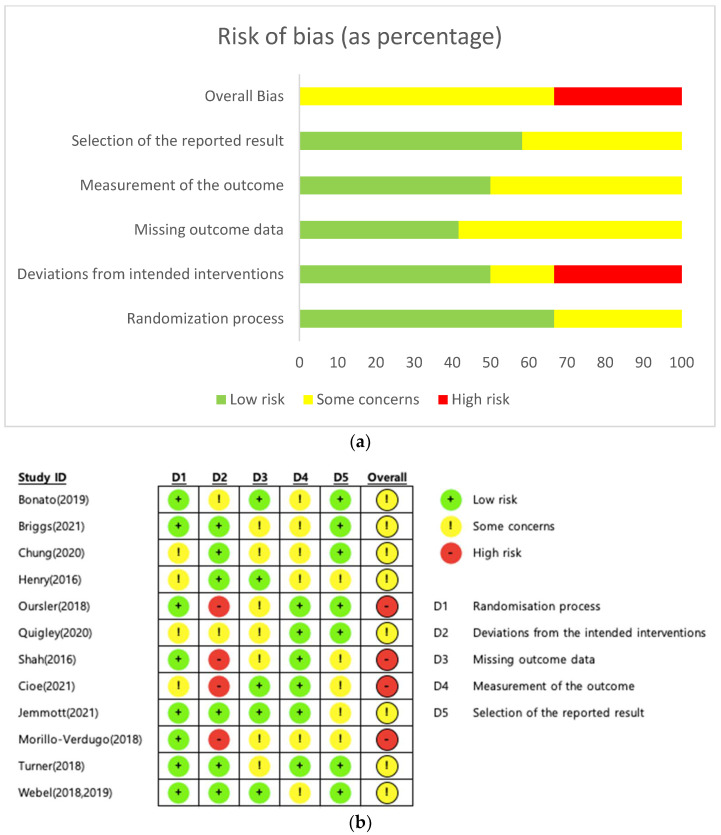
Risk-of-bias graphs for the studies included for the meta-analysis and systematic review. (**a**) Risk-of-bias as a percentage; (**b**) risk-of-bias summary [16,17,18,19,27,33,34,35,36,37,38,39,40].

Figure 2 shows the risk-of-bias results for the included studies. Among 12 studies, the overall bias of 8 studies was classified as “having some concerns” [16,18,27,33,34,35,36,37,38], and the overall bias of 4 studies was classified as “high risk” [17,19,39,40]. A high risk-of-bias was identified by deviation from the intended interventions, as participants were not blinded to the intervention allocation. In addition, these deviations from the intended interventions were not balanced between the intervention and comparison groups. Between the intervention and comparison groups, there were missing outcome data, as well as differences in attrition rates and reasons for dropout, thus leading to a high risk-of-bias.

### 3.3. Study Characteristics

Of the 12 studies, two-thirds were conducted in the United States (*n* = 8), while the others were conducted in Canada (*n* = 1), Hong Kong (*n* = 1), Italy (*n* = 1), and Spain (*n* = 1). Among the studies performed in the United States, the articles by Webel et al. (2018) and Webel et al. (2019) were derived from one study [18,38]. The sample size of the studies ranged from 16 to 302 participants. Most studies included both men and women (*n* = 10), with two studies including only men [17,35]. Table 1 shows the detailed characteristics of each study.

### 3.4. Study Participants’ Characteristics

Three of the included studies targeted physically inactive sedentary adults living with HIV [16,17,33]. In five studies, interventions were targeted at people with HIV and other comorbidities presenting certain symptoms or diagnoses such as cardiovascular diseases [18,38,40], neurocognitive impairment [34], mild-to-moderate functional limitations [19], and chronic lower back or lower extremity pain [37]. In one study, participants were specifically African American middle-aged men with HIV [35], while in three other studies participants included adults living with HIV not limited to specific conditions [27,36,39].

### 3.5. Intervention Characteristics

Details concerning the intervention characteristics of each study is presented in Table 2. Seven of the twelve studies provided interventions focused only on physical activity; among these, five studies provided interventions in which participants performed physical activities face-to-face: high-intensity aerobic exercise [16,17,27], group-based moderate-intensity aerobic and resistance exercise [33], and group-based yoga classes [36]. The other two studies provided interventions to promote physical activity. The first study monitored step count using a pedometer and provided feedback about the physical activity using text messages [34], while the second one provided counseling on overcoming barriers that affected participants’ physical activity and setting physical activity goals [19].

Five of the twelve studies implemented interventions that combined physical activity with other health-related content. In two studies, education, interactive activities, and discussion about health promotion including physical activity were conducted [18,35,38]. Another two studies aimed at decreasing the cardiovascular disease risk in people living with HIV [39,40]. Among these, one study provided personalized feedback and motivational interviewing for cardiovascular disease risk factor awareness and behavior change, which included physical activity [39]. The other study performed a structured pharmaceutical care intervention to achieve pharmacotherapeutic objectives related to cardiovascular risk and provided health behavior recommendations for cardiovascular risk prevention [40]. Finally, the remaining study provided lectures and supplies for stretching and strengthening exercises for chronic pain self-management [37].

The interventions in the 12 studies were delivered as follows. Six studies provided face-to-face physical activity interventions [16,17,18,33,35,36,38]. Five studies supplemented the face-to-face physical activity interventions with additional strategies, such as phone calls [19,37], text messages [39,40], and a mobile application [27]. One study implemented a physical activity intervention using only mobile phones [34].

The length of the physical activity interventions ranged from 3 to 48 weeks. Eight studies provided interventions for at least 12 weeks [16,17,19,27,34,36,37,40], while four studies provided interventions for 8 weeks or less [18,33,35,38,39]. Active control groups were used for comparison in seven studies, while the other five made use of comparison groups that implemented usual care (*n* = 3), a wait-list control (*n* = 1), or assessment-only (*n* = 1).

### 3.6. Physical and Phycological Health Outcomes

Physical and psychological health outcomes that were assessed in the included studies were categorized into the following five outcomes: walking capacity, cardiorespiratory fitness, body composition and weight, metabolic parameters, and psychological profiles. The 6-min walk test [17,19,33,37] and gait speed [19,36] were used to assess walking capacity. The peak oxygen uptake (VO_2_peak) [16,17,18,32] and time spent on a treadmill [14,15] were used to assess cardiorespiratory fitness. Body fat percentage [16,17,32], fat mass [16,17], and weight [16,17,38] were grouped in the outcome of body composition and weight. The metabolic parameters included total cholesterol, low-density lipoprotein (LDL) cholesterol, high-density lipoprotein (HDL) cholesterol, and triglycerides, which were assessed in four studies, respectively [16,17,32,40]. Depression [19,32,36] and health-related quality of life [19,33,36] were categorized into the outcome of psychological profile (Table 3).

### 3.7. Effects of Physical Activity Interventions

**Figure 3:**
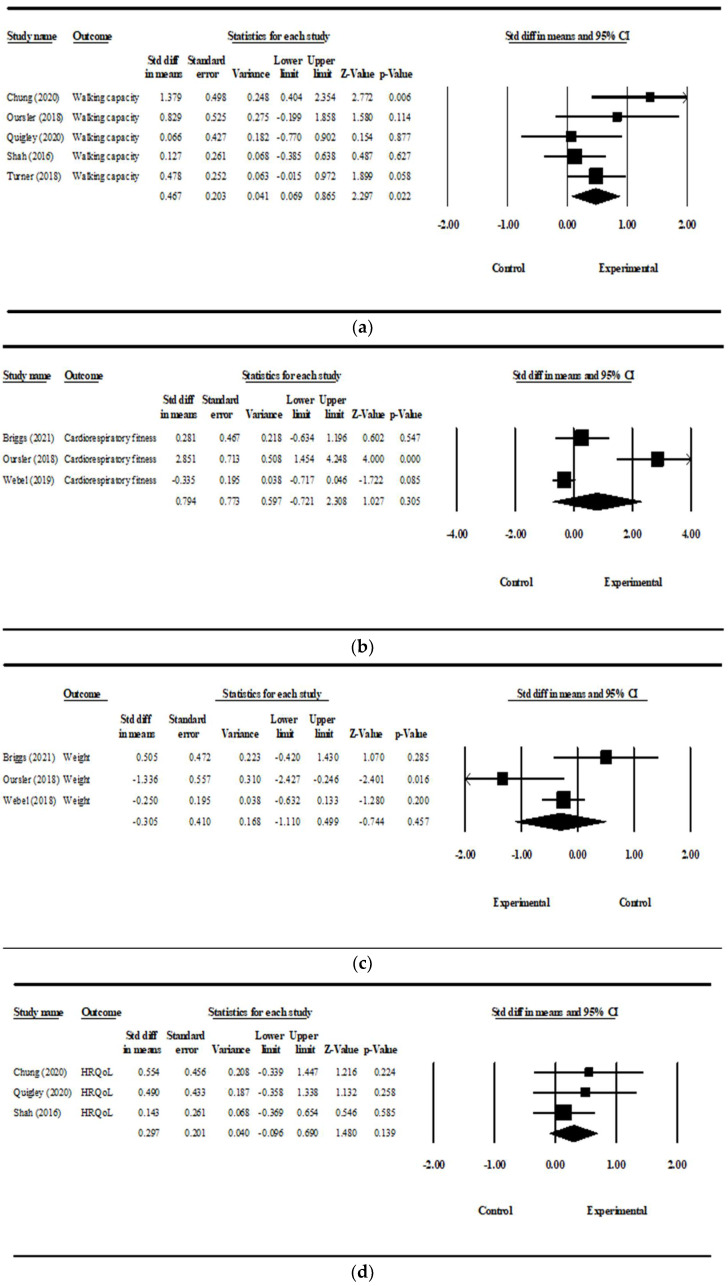
Forest plots showing the effect sizes of physical activity interventions. (**a**) Effect size on walking capacity [17,19,33,36,37]; (**b**) effect size on cardiorespiratory fitness [16,17,18]; (**c**) effect size on weight [16,17,38]; (**d**) effect size on health-related quality of life [19,33,36].

The data from eight articles [16,17,18,19,33,36,37,38] derived from seven studies were used in the meta-analyses. Meta-analyses of physical activity interventions on walking capacity, cardiorespiratory fitness, weight, and health-related quality of life were conducted. Forest plots depicting the effect sizes by type of outcomes are shown in Figure 3. The effect size of physical activity interventions compared with the control group on walking capacity was medium (Cohen’s *d*: 0.467; 95% CI [0.069, 0.865]), with moderate heterogeneity (I^2^ = 36.786%). The effect size on cardiorespiratory fitness (Cohen’s *d*: 0.794; 95% CI [−0.721, 2.308]; I^2^ = 89.591%), on weight (Cohen’s *d*, −0.305; 95% CI [−1.110, 0.499]; I^2^ = 68.602%), and on health-related quality of life (Cohen’s *d*, 0.297; 95% CI [−0.096, 0.690]; I^2^ = 0%) were not significant.

### 3.8. Sensitivity Analysis and Publication Bias

The sensitivity analysis on walking capacity was shown in Table 4. Although Chung et al. (2020) and Shah et al. (2016) had greater impacts on the pooled effect size than the other three studies, the effect sizes after eliminating the two studies one by one were not changed significantly and were still significantly positive. Therefore, the result of the meta-analysis on walking capacity was relatively stable. 

For the results of sensitivity analyses on cardiorespiratory fitness, weight, and health-related quality of life, the effect sizes after excluding each of the pooled studies one by one were not changed significantly and remained non-significant; therefore, they indicated that the results of meta-analyses on cardiorespiratory fitness, weight, and health-related quality of life were relatively stable. 

The funnel plot for walking capacity indicated there is symmetry in the distribution of effect sizes. In addition, Egger’s test on walking capacity showed no statistically significant publication bias (*p* = 0.317). Although the funnel plot for cardiorespiratory fitness showed an asymmetrical distribution, Egger’s test showed no significant publication bias (*p* = 0.261). The funnel plot for weight showed symmetrical distribution, and Egger’s test also showed no significant publication bias (*p* = 0.894). Regarding health-related quality of life, an asymmetrical distribution of the funnel plot and a significant bias in Egger’s test (*p* = 0.018) were found. 

## 4. Discussion

We conducted a systematic review and meta-analysis regarding physical activity interventions on physical and psychological health outcomes among older adults living with HIV. This study was differentiated from previous systematic reviews and meta-analyses [20,21,23,24] by specifically targeting older adults living with HIV. Our results showed that physical activity interventions had a significant effect on walking capacity. However, our meta-analyses did not support significant effects on cardiorespiratory fitness, weight, and health-related quality of life.

Our meta-analysis found that physical activity interventions had a significant effect on the outcome of walking capacity, including 6-min walk and gait speed. Among the studies included in this meta-analysis, the studies by Chung et al. [33] and Oursler et al. [17] showed large effect sizes. Chung et al. [33] implemented an intervention of moderate-intensity aerobic and resistance exercise lasting 45 min twice a week, and Oursler et al. [17] implemented an intervention of high-intensity aerobic exercise lasting 20–45 min three times a week. A previous scoping review reported that moderate-to-high intensity aerobic exercise and combined aerobic and resistance training were effective for enhancing the walking capacity of older adults living with HIV [41]. Therefore, we suggest that health care providers should consider providing physical activity interventions such as combined aerobic and resistance training or high-intensity aerobic exercise for improvement of walking capacity in older adults living with HIV.

In this review, physical activity interventions had no significant effect on the outcome of cardiorespiratory fitness, including VO_2_ peak and time on treadmill. Among the three studies pooled in the meta-analysis on cardiorespiratory fitness, one study providing supervised high-intensity aerobic exercise for 16 weeks reported a significant improvement of VO_2_ peak in older adults living with HIV [17]. Meanwhile, another study pooled in the meta-analysis that provided education for lifestyle behavior change including physical activity, reported no significant improvement of VO_2_ peak [18]. Thus, education for physical activity may not lead to an increase in actual physical activity performance and improved cardiorespiratory fitness compared with aerobic exercise under supervision. Previous studies have suggested that the most important aspect in improving cardiorespiratory fitness is to increase physical activity in sufficient quantity and intensity [18,20]. Therefore, we suggest that health care providers should provide physical activity interventions that can lead to sufficient increase in the quantity of physical activity to improve the cardiorespiratory fitness of older adults living with HIV.

This review showed no significant effect of physical activity interventions on weight. According to the gold standard behavioral obesity treatment suggested by Thomas et al., it is necessary to provide interventions for dietary intake as well as for physical activity to achieve weight loss [42]; specifically, establishing goals for weight loss, dietary intake, and physical activity, and self-monitoring and feedback on them are needed for weight loss [42]. In this review, the included studies that assessed body composition and weight as outcomes focused either on improving physical activity without implementing interventions for dietary intake [16,17,27] or on providing short-term education on physical activity and healthy diet [38]. Thus, the nonsignificant effect on body composition and weight in this review may be explained by the lack of focus on dietary interventions in the included studies. Future studies should incorporate elements such as nutritional education, tailored diet planning, and monitoring of dietary intake into physical activity interventions to improve outcomes related to body composition and weight.

The included four studies assessed metabolic parameters including total cholesterol, LDL cholesterol, HDL cholesterol, and triglycerides [16,17,27,40]. People living with HIV have a higher risk of cardiovascular disease than uninfected people [43], particularly if they have high total cholesterol and triglycerides and were older age [44]. Therefore, monitoring metabolic parameters and providing interventions to improve these parameters should be considered for older adults living with HIV. Since this review included only two studies reporting sufficient data to calculate the effect size on metabolic parameters [16,40], we could not quantitatively confirm the effects of physical activity interventions on metabolic parameters among older adults living with HIV. Further research assessing metabolic parameters as outcomes are required to determine the effects.

Our review could not confirm that physical activity interventions had significant effects on psychological profiles, including health-related quality of life and depression. A previous review reported that the combined aerobic and resistance exercise was the most effective exercise type for improving health-related quality of life for people living with HIV [45]. In addition, a different review reported that there were large effects on depression improvement in people living with HIV with an aerobic exercise intervention, three or more times per week, supervised by a professional [23]. However, because our review found that the evidence on the effects on depression and health-related quality of life for older persons was scarce, future studies are needed to determine the effects of physical activity interventions on depression and health-related quality of life for this population.

To evaluate walking capacity among older adults living with HIV, four studies used the 6-min walk as a measurement [17,19,33,37]. The 6-min walk is a test in which a technician measures the distance a participant can walk in six minutes according to a standardized protocol, and it can be performed quickly and safely by older adults [46]. Therefore, we suggest the 6-min walk test as a useful test to measure walking capacity in older adults living with HIV. Recent studies have suggested that the 6-min walk test using wearable sensors and a mobile phone can measure a patient’s detailed movement and mobility, therefore may be more relevant to the patient’s daily activity than the traditional in-clinic standardized test [47,48]. Thus, further studies are needed to assess the usability and validity of the test incorporating these technologies in older adults living with HIV.

As a result of the risk-of-bias evaluation, 4 studies out of 12 were classified as “high risk”; the major cause was the failure to blind participants in the allocation and/or assessment process. Due to the nature of physical activity interventions, it is not easy to blind the allocation process; however, if blinding is not achieved, the true effect of interventions cannot be estimated [49]. Therefore, high-quality RCT protocols, that secure blinding allocation through comparison with an active control group, are essential to investigating the true impact of physical activity interventions.

This review has several limitations. First, this review included only publications written in English. Because this review did not include grey literature, it may increase the risk of publication bias. In the results of assessing publication bias in meta-analyses, Egger’s tests for publication bias were not statistically significant in the outcomes of walking capacity, cardiorespiratory fitness, and weight, except for health-related quality of life. Second, this review included 13 articles derived from 12 studies, and our meta-analyses included 8 articles derived from 7 studies. Thus, this review has a limitation of the small number of included studies, therefore more RCTs are required to determine effects of physical activity interventions among older adults living with HIV. Third, most of the included studies were conducted in the United States. Therefore, generalizing our results globally should be done with caution.

## 5. Conclusions

This study provides an integrated perspective on the current evidence regarding physical activity interventions for people living with HIV aged ≥50 years. This study demonstrates that physical activity interventions are effective in improving patients’ outcome of walking capacity including 6-min walk and gait speed. The high-intensity aerobic exercise and the intervention of moderate-intensity aerobic and resistance exercise had a significant effect on the improvement of walking capacity in older adults living with HIV. This review could not confirm the significant effects on cardiorespiratory fitness, body composition and weight, metabolic parameters, and psychological profiles. We suggest using supervised physical activity interventions that can directly increase exercise performance to improve cardiorespiratory fitness. In addition, to improve body composition and weight, integrated interventions of physical activity and dietary interventions are suggested. Further RCTs with a larger scale are required to determine the effects of physical activity interventions for aged people living with HIV on outcomes of cardiorespiratory fitness, body composition and weight, metabolic parameters, and psychological profile.

## Figures and Tables

**Table 1:** Characteristics of studies selected for the systematic review and meta-analysis on people aged ≥50 years with HIV.

First Author (Year)	Country	Target Population	Age (Mean, Years)		Sample Size (Recruitment)		Sex
			IG	CG	IG	CG	
**Interventions focused only on physical activity**							
Bonato (2020) [27]	Italy	Adults living with HIV	52.0 ^a^	50.0 ^a^	20	18	82.4% male
Briggs (2021) [16]	US	Sedentary adults living with HIV who were 50 years and older	63.4	60.1	13	13	94.7% male
Chung (2020) [33]	Hong Kong	Physically inactive adults living with HIV	66.5	70.3	11	10	75% male
Henry (2016) [34]	US	Adults with HIV-associated neurocognitive impairment diagnosis	49.6	51.8	11	10	85.7% male
Oursler (2018) [17]	US	Sedentary adults living with HIV who were 50 years and older	57.4	57.4	11	11	100% male
Quigley (2020) [36]	Canada	Adults living with HIV	50.7	60.2	11	11	68.2% male
Shah (2016) [19]	US	Adults living with HIV with mild-to-moderate functional limitations	54.6	56.2	33	34	61.0% male
**Interventions involving physical activity**							
Cioe (2021) [39]	US	Adults living with HIV	48.8	53.9	19	21	60.0% male
Jemmott (2021) [35]	US	African American middle-aged men living with HIV	53.6	54.2	152	150	100.0% male
Morillo-Verdugo (2018) [40]	Spain	Adults living with HIV receiving ART with at least 1 drug for the treatment of CVD or diabetes and at a moderate or high risk of CVD	53.6 ^b^	53.6 ^b^	26	33	90.6% male
Turner (2018) [37]	US	Adults living with HIV with chronic lower back or lower extremity pain, and who were prescribed opioid analgesics	56.9	56.2	53	58	45.0% male
Webel (2018) [38]; Webel (2019) [18]	US	Adults living with HIV at high risk for developing CVD	52.3	53.3	54	53	64.5% male

Note. IG—Intervention Group; CG—Control Group; ART—Antiretroviral Treatment; CVD—Cardiovascular Disease. a—this study only reported the median age of participant groups. b—this study only reported the mean age of the total participants.

**Table 2:** Characteristics of physical activity interventions implemented in studies on people living with HIV aged 50 years or older.

First Author (Year)	Title of Intervention	Intervention Description	Mode of Delivery	Period; Time/Session; Frequency	Provider of Intervention	Comparison Condition	Intervention Adherence
**Interventions focused only on physical activity**							
Bonato (2020) [27]	A mobile application and aerobic exercise intervention (Progetto appfitness)	(1) Weeks 1–4, with direct coach supervision, with training intensity set at 60–70% of maximal heart rate; (2) Weeks 5–16, without coach supervision, at a training intensity of 70–80% of maximal heart rate, which is expected to improve aerobic fitness, (3) a weekly notification of training plan and prescription through the mobile app	Face-to-face and mobile application	16 weeks; 1 h; 3 times/week	Professional coach	Aerobic exercise excluding mobile application use	(1) Coach supervision (weeks 1–4): 100%; (2) autonomous training (weeks 5–16): 60% (median)
Briggs (2021) [16]	High-intensity interval AEX combined with resistance training	(1) Weeks 1–4, participants started at 50–60% HRR for 15 min and were progressed until they reached at least 30 min at 60% HRR; (2) weeks 5–16, the intensity was increased as tolerated to 70–80% HRR, and duration was titrated to the goal of 30–40 min of high-intensity AEX	Face-to-face	16 weeks; 15–45 min; 3 times/week	Exercise physiologist	Unchanged physical activity level and then delayed high-intensity interval training combined with resistance training	Median attendance rate: 89%
Chung (2020) [33]	Supervised exercise	Moderate-intensity exercise (maintained 50–70% of heart rate) combined with aerobic and resistance training in the form of group-based training sessions for two to three participants	Face-to-face	8 weeks; 45 min; 2 times/week	Physiotherapist	Being advised to continue routine daily activities, and self-motivated exercise was allowed	96.3% program attendance rate to completion
Henry (2016) [34]	iSTEP (SMS/MMS intervention)	Interactive and personalized daily text messages, step count monitoring with a pedometer, text, and MMS feedback of physical activity changes over time, message reminders tailored to each participant’s barriers and preferred activities, and weekly goal-setting	Mobile phone	16 weeks; not reported; 3 times/day	N/A	Text messages 3 times a day throughout the 16 weeks about HIV symptoms and mood	(1) Responding to text messages: 89%; (2) reporting the daily step counts: 92%
Oursler (2018) [17]	High-intensity aerobic exercise	Starting with aerobic exercise training for 20–30 min at 50–60% of HRR, progressively increasing by 10% of HRR each week so that within 5–7 weeks the aerobic exercise sessions lasted 30–45 min at 70–85% of HRR and at the end of the 16 weeks lasted 40–45 min at 75–90% of HRR	Face-to-face	16 weeks; 20–45 min; 3 times/week	Exercise physiologist	Moderate-intensity aerobic exercise	Mean attendance rate: 89%
Quigley (2020) [36]	Yoga intervention	Group-based yoga classes with classes consisting of seated meditation, breathing exercises, shoulder, neck, and back stretches, and sun salutations (either seated or standing), standing poses, balance poses, abdominal and back-bend poses, and cool-down stretches and final rest	Face-to-face	12 weeks; 60 min; 3 times/week	Yoga instructor	Usual care	Mean attendance rate: 82%
Shah (2016) [19]	Physical activity counseling intervention based on self-determination theory	Counseling program for personal decision making, while giving the support needed to ensure proper education: (1) the first counseling session (60-min): understanding participants’ interests, values, and behaviors and encouraging them to discuss barriers to physical activity and solutions to overcoming them; (2) autonomy supportive sessions: follow-up telephone counseling sessions to facilitate setting appropriate physical activity goals	Face-to-face and telephone calls	12 weeks; 60 min for 1st session, 15–30 min for phone calls; 2 times/month	Physician and mental health therapist, physical therapist	Usual care	93% of participants participated in at least four out of six counseling sessions
**Interventions combining physical activity with other health-related contents**							
Cioe (2021) [39]	CVD-PRAI	Personalized feedback and motivational interviewing: (1) Session 1, discussion of CVD risk and modifiable risk factors, advice for behavior change and setting goals, and providing related literature; (2) Session 2, summary of the prior session, review of goals, addressing barriers to change, and discussion of strategies for maintaining long-term behavior change	Face-to-face and mobile phone (text message)	4 weeks; 45 min; 2 sessions; daily text message during week 1, weekly during week 2–4	Nurse	Brief health education to improve heart-healthy behaviors	90% of participants completed all sessions
Jemmott (2021) [35]	“Men Together Making a Difference” health promotion intervention	Brainstorming, educational games, and interactive activities including physical exercise and videos, to increase adherence to guidelines for physical activity, diet, and colon cancer screening	Face-to-face	3 weeks; 1 h; 3 times/week	Trained facilitator	One 60-min small group session	100% of participants attended 1st week and 97% attended 2nd and 3rd week
Morillo-Verdugo (2018) [40]	Structured pharmaceutical care intervention	Intensive pharmaceutical care to reduce cardiovascular risk: (1) pharmacotherapeutic follow-up of all medication taken by the patient to work toward achieving pharmacotherapeutic objectives related to cardiovascular risk; (2) recommendations for improving diet, exercise, and smoking cessation; (3) providing leaflets on cardiovascular risk prevention and an individual motivational interview; (4) periodic contacts by sending text messages with content related to healthy living habits and health promotion	Face-to-face, leaflet, and mobile phone (text message)	48 weeks; not reported; 5 visits/48 weeks; weekly text message during week 1–4, then periodically until the end of the follow-up period	Pharmacist	Unchanged physical activity level	Not reported
Turner (2018) [37]	“Living Better Beyond Pain" program (chronic pain self-management program)	Pain self-management program: (1) one-on-one lectures for pain self-management topics and exercise demonstration; (2) providing additional materials included activity logs with personal goals, program DVDs (walking exercises, self-massage techniques), exercise mats, tennis balls for massage, and multi-pronged self-massage tools	Face-to-face and telephone	24 weeks; 30–45 min; 6 times/6 months; at least one phone call between visits	Health educator	Pain self-management program in the community setting	62.1% of participants completed all measures; 36% attended all of meetings
Webel (2018) [38]; Webel (2019) [19]	Lifestyle behavior intervention (“System CHANGE")	Group sessions for: (1) behavior change techniques to achieve a specific participant-defined goal to improve lifestyle behaviors (physical activity and diet); (2) education that emphasized a diet consisting of low-energy-density foods through increased fresh fruits, vegetables, and whole grains; (3) discussion about the types of physical activity, issues that may interfere with sufficient activity, and techniques to modify the participants’ physical environment to encourage activity and eating a healthy diet; (4) discussion on how to incorporate healthy eating and physical activity into the participant’s daily routine	Face-to-face	6 weeks; 1 h; 1 time/week	Health educator	Pamphlet that contained information on healthy eating and physical activity	90% of the participants attended at least half of the sessions and 60% attended at least 5 sessions

Note. HRR—Heart Rate Reserve, AEX—Aerobic exercise; SMS—Short Message Service; MMS—Multimedia Message Service; N/A—Not Applicable; CVD—Cardiovascular Diseases; PRAI—Perceived Risk Awareness Intervention.

**Table 3:** Health outcomes assessed in the included studies.

Health Outcomes	How Assessed (Studies That Assessed the Outcomes)
**Walking capacity**	
6-min walk	Measuring the distance a participant walked in six minutes for evaluaton of walking capacity [17,19,33,37]
Gait speed	Measuring the time it takes to walk a specific distance as quickly and safely as possible for evaluation of dynamic balance performance [19,36]
**Cardiorespiratory fitness**	
VO_2_ peak	Measuring oxygen uptake at peak exercise performance during graded exercise test by treadmill [16,17,27] or bicycle ergometer [18] for evaluation of cardiorespiratory fitness
Time on treadmill	Measuring total exercise duration of a graded exercise treadmill test for evaluation of exercise endurance [16,17]
**Body composition and weight**	
Body fat percent	Calculating total fat mass divided by total body mass after measuring fat mass and lean mass by dual-energy X-ray absorptiometry [16,17] or bioimpedentiometry [27]
Fat mass	Measuring fat mass by dual-energy X-ray absorptiometry [16,17]
Weight	Measuring body weight using a scale [16,17,38]
**Metabolic parameters**	
Total cholesterol	Testing total cholesterol by laboratory analysis after overnight fasting and blood draw [16,17,27,40]
LDL cholesterol	Testing low-density lipoprotein (LDL) cholesterol by laboratory analysis after overnight fasting and blood draw [16,17,27,40]
HDL cholesterol	Testing high-density lipoprotein (HDL) cholesterol by laboratory analysis after overnight fasting and blood draw [16,17,27,40]
Triglycerides	Testing triglyceride by laboratory analysis after overnight fasting and blood draw [16,17,27,40]
**Psychological profile**	
Depression	Assessing depressive symptoms using self-reported questionnaires such as Beck Depression Inventory-II [19], Psychological Monitoring of Overtraining and Staleness [27], and Hospital Anxiety and Depression Scale [36]
Health-related quality of life	Assessing health-related quality of life using self-reported questionnaires such as Short-Form Health Survey (36-item) [19,33], and Medical Outcomes Survey-HIV [36]

**Table 4:** Sensitivity analysis for walking capacity excluding each study one by one.

Study Omitted	Pooled Estimate	95% Confidence Interval		*p* Value
		Lower	Upper	
Chung (2020) [33]	0.323	0.011	0.634	0.042
Oursler (2018) [17]	0.426	−0.028	0.879	0.066
Quigley (2020) [36]	0.560	0.092	1.028	0.019
Shah (2016) [19]	0.607	0.130	1.085	0.013
Turner (2018) [37]	0.507	−0.076	1.090	0.088
Pooled (random effect)	0.467	0.069	0.865	0.022

## Data Availability

The original contributions generated for the study are included in the article/supplementary material, further inquiries can be directed to the corresponding author.

## Supplementary Materials

The following supporting information can be downloaded at: https://www.mdpi.com/article/10.3390/ijerph19148439/s1, Table S1: Search strategies.
