# Supplementary material for: Effects of Physical Activity Interventions on Health Outcomes among Older Adults Living with HIV: A Systematic Review and Meta-Analysis
Source: Int J Environ Res Public Health. 2022 Jul 11;19(14):8439. doi: 10.3390/ijerph19148439 (PMC9317429; doi:10.3390/ijerph19148439)
Supplement: Supplementary file 1 [file ijerph-19-08439-s001.zip › ijerph-1780221-supplementary.pdf]

Supplementary file

Table S1. Search Strategies

No.	Search Strategies	Results	Remarks
**Pubmed**			
1	"HIV"[tiab] OR "Acquired Immunodeficiency syndrome"[tiab] OR "HIV"[mesh] OR "Acquired Immunodeficiency syndrome"[mesh]	387,106	
2	"Intervent*"[tiab] OR "Program*"[tiab] OR "Treat*"[tiab] OR "Therap*"[tiab] OR "Program evaluation"[mesh] OR "Therapeutics"[mesh]	11,301,695	
3	"Physical activity"[tiab] OR "Exercise"[tiab] OR "Exercise"[mesh]	471,760	
4	"Randomized controlled trial"[pt] OR "Controlled clinical trial"[pt] OR "Randomized controlled trial*"[tiab] OR "Trial"[tiab] OR "Random*"[tiab] OR "Single blind*"[tiab] OR "Double blind*"[tiab] OR "Triple blind*"[tiab] OR "Randomized Controlled Trials as topic"[mesh] OR "Randomized Controlled Trial"[pt] OR "Controlled Clinical Trials as topic"[mesh] OR "Controlled Clinical Trial"[pt] OR "Random Allocation"[mesh] OR "Single-Blind Method"[mesh] OR "Double-Blind Method"[mesh]	1,886,966	
5	#1 AND #2 AND #3 AND #4	334	
6	#1 AND #2 AND #3 AND #4	139	Filters: English, age +45, human
**Total**		139	
**No.**	**Search Strategies**	**Results**	**Remarks**
**Embase**			
1	('hiv':ab,ti OR 'acquired immunodeficiency syndrome':ab,ti) AND ('interventot*':ab,ti OR 'program*':ab,ti OR 'treat*':ab,ti OR 'therap*':ab,ti OR 'program evaluation':ab,ti) AND ('physical activity':ab,ti OR 'exercise':ab,ti) AND ('randomized controlled trial':ab,ti OR 'controlled clinical trial':ab,ti OR 'randomized controlled trial*':ab,ti OR 'trial':ab,ti OR 'random*':ab,ti OR 'single blind*':ab,ti OR 'double blind*':ab,ti OR 'triple blind*':ab,ti)	303	
2	('hiv'/exp OR 'acquired immunodeficiency syndrome'/exp) AND ('interventot*' OR 'program*' OR 'treat*' OR 'therap*' OR 'program evaluation'/exp) AND ('physical activity'/exp OR 'exercise'/exp) AND ('randomized controlled trial'/exp OR 'controlled clinical trial'/exp OR 'randomized controlled trial*' OR 'trial'/exp OR 'random*' OR 'single blind*' OR 'double blind*' OR 'triple blind*')	197	
3	#1 OR #2	398	
4	#3 AND 'human'/de AND ([aged]/lim OR [middle aged]/lim)	81	Filters: aged, middle aged, human
**Total**		81	
**No.**	**Search Strategies**	**Results**	**Remarks**
**CINAHL**			
1	AB "HIV" OR "Acquired human immunodeficiency syndrome" OR (MH "HIV infections")	101,545	

2	"intervent*" OR "program*" OR "treat*" OR "therap*" OR (MH "program evaluation") OR (MH "nursing interventions") OR (MH "experimental studies") OR (MH "intervention trials")	3,028,027	
3	"physical activity" OR "exercise" OR (MH "exercise") OR (MH "resistance training") OR (MH "therapeutic exercise")	242,337	
4	"randomized controlled trial*" OR "controlled trial*" OR "controlled clinical trial" OR "trial*" OR "random*" OR "single blind*" OR "double blind*" OR "triple blind*" OR (MH "randomized controlled trials") OR (MH "clinical trials")	710,683	
5	AB #s1 and #s2 and #s3 and #s4	92	
7	AB #s1 and #s2 and #s3 and #s4	16	Filters: English, age 45+
**Total**		16	

No.	Search Strategies	Results	Remarks
**Cochrane Library CENTRAL**			
1	"HIV" OR "Acquired Immunodeficiency syndrome"	29,933	
2	[Mesh] HIV	3,188	
3	[Mesh] Acquired Immunodeficiency syndrome	2,005	
4	#1 OR #2 OR #3	29,933	
5	"Intervent*" OR "Program*" OR "Treat*" OR "Therap*" OR "Program evaluation" OR "Therapeutics"	219,841	
6	[Mesh] program evaluation	6,446	
7	[Mesh] therapeutics	318,868	
8	#5 OR #6 OR #7	489,951	
9	"Physical activity" OR "Exercise"	127,783	
10	[Mesh] Exercise	26,876	
11	#9 OR #10	130,778	
12	"Randomized controlled trial*" OR "Trial" OR "Random*" OR "Single blind*" OR "Double blind*" OR "Triple blind*"	1,426,624	
13	[Mesh] Randomized Controlled Trials as Topic	15,075	
14	[Mesh] Controlled Clinical Trials as Topic	15,229	
15	[Mesh] Random Allocation	20,654	
16	[Mesh] Single-Blind Method	22,262	
17	[Mesh] Double-Blind Method	143,967	
18	#12 OR #13 OR #14 OR #15 OR #16 OR #17	1,426,881	
19	#4 AND #8 AND #11 AND #18	385	
20	#4 AND #8 AND #11 AND #18	215	
**Total**		215	
